# Comprehensive dosimetric commissioning of proton minibeam radiotherapy on a single gantry proton system

**DOI:** 10.3389/fonc.2024.1421869

**Published:** 2024-07-19

**Authors:** Yuting Lin, Wangyao Li, Aoxiang Wang, Daniel Johnson, Gregory N. Gan, Hao Gao

**Affiliations:** ^1^ Department of Radiation Oncology, University of Kansas Medical Center, Kansas City, KS, United States; ^2^ Department of Biomedical Engineering, Huazhong University of Science and Technology, Wuhan, China

**Keywords:** proton therapy, spatially fractionated radiotherapy, proton mini-beam, small field dosimetry, film dosimetry, proton dosimetry

## Abstract

**Background:**

Proton minibeam radiation therapy (pMBRT) can deliver spatially fractionated dose distributions with submillimeter resolution. These dose distributions exhibit significant heterogeneity in both depth and lateral directions. Accurate characterization of pMBRT doses requires dosimetry devices with high spatial resolution and a wide dynamic range. Furthermore, the dependency of dosimetric measurements on Linear Energy Transfer (LET), as observed in conventional proton therapy, is also present in pMBRT depth dose measurements.

**Purpose:**

This work demonstrates the process of performing comprehensive dosimetric measurements to characterize the pMBRT collimator on a clinical single-gantry proton machine, utilizing commercially available dosimetry devices.

**Methods:**

The minibeam collimator is designed to be mounted on the clinical nozzle as a beam-modifying accessory. Three collimators, each with a slit opening of 0.4 mm, are thoroughly evaluated. The center-to-center (c-t-c) distances of the slits for these collimators are 2.8 mm, 3.2 mm, and 4.0 mm, respectively. High spatial resolution dosimetry devices are essential for PMBRT dose characterizations. To meet this requirement, two-dimensional (2D) dose measurement devices, Gafchromic films, are used to measure lateral profiles at various depths. Films are also used for depth dose profile measurements in solid water. Additionally, high-resolution point dose detectors, microDiamond, and Razor diode detectors are employed for lateral profile measurements at various depths. Percent depth dose (PDD) measurements of pMBRT in solid water, with various proton energies, collimators, and air gaps, are performed using Gafchromic films. The film’s LET dependency for proton beams is corrected to ensure accurate pMBRT PDD measurements. The Monte Carlo simulation tool TOPAS is utilized to compare and validate all experimental measurements.

**Results:**

At depths where LET is not a concern, film dose measurements were consistent with microDiamond and Razor diode point measurements. The point detectors need to be orientated with the thin side aligned to the incoming beam. Comparison of the lateral dose profiles extracted from TOPAS simulations, films, microDiamond, and Razor diode detectors shows a passing rate exceeding 98% in 1D gamma analysis at 3% 0.1 mm criteria.However, when the microDiamond detector is orientated to face the pMBRT beam, its spatial resolution may not be sufficient to capture the peak and valley dose accurately. Nevertheless, an accuracy within 2% can still be achieved when comparing the average dose. The PDD measurements show that the peak valley dose ratio (PVDR) of pMBRT can be altered at different depths with different air gaps using the same collimator or different collimators of different c-t-c distances.

**Conclusion:**

Our study demonstrates that comprehensive dose measurements for pMBRT can be conducted using standard clinical dose measurement devices. These measurements are indispensable for guiding and ensuring accurate dose reporting in pre-clinical studies using the pMBRT technique.

## 1 Introduction

Spatially fractionated radiotherapy (SFRT) delivers spatially modulated heterogeneous radiation dose characterized by the co-existence of both high-dose peaks and low-dose valleys (1–4). Preclinical and clinical studies have shown that various forms of SFRT can substantially increase normal tissue dose tolerance while maintaining or improving tumor control efficacy (1, 4–7). The precise biological mechanisms behind SFRT represent a subject of ongoing studies but are not yet fully understood (2, 4, 8). The minibeam modality of SFRT is characterized by a submillimeter beamlet width, which has been shown to increase organ at risk (OAR) tolerances in pre-clinical studies (7, 9–14). The potential clinical significance of the minibeam form of SFRT includes the following key aspects. Firstly, the minibeam achieves the smallest beamlet size that remains deliverable using existing clinical radiation therapy devices. This applies to both photon and proton modalities, in contrast to the microbeam techniques, where the beam width is typically less than 0.1 mm. Secondly, the small slit size and slit distances of minibeam SFRT make it more suitable for pre-clinical studies compared to GRID/LATTICE techniques, which typically have centimeter-scale beam opening and beamlet distances. Mini-beam pre-clinical studies enable biologists to explore the underlying biological mechanism for SFRT. Finally, the smaller scale of minibeam SFRT also enables the treatment of smaller tumors in humans using this technique.

The proton beam can deliver the most radiation dose to the tumor target and stop completely at the distal end of the treatment depth (15, 16). In contrast, photon treatment continuously deposits radiation dose until exiting the body. Combining the minibeam form of SFRT with proton treatment modality results in proton minibeam radiation therapy (pMBRT), representing a synergistic integration of both techniques (17–21). This pMBRT approach also holds significant translational potential for implementation in clinical proton therapy facilities, facilitating pre-clinical and translational studies.

We have implemented the first pMBRT system at a single gantry proton facility (22). The system features a plug-and-play design, allowing efficient and reproducible setup within minutes. The pMBRT collimator, machined using 6.5 cm thick brass, fits into the aperture slot of the clinical snout. It can be pre-assembled, adjusted, and made ready for use on the day of the experiment. The proton minibeam pattern is generated by irradiating a collimator with a pencil beam that is uniformly scanned across the collimator entrance. This work expands the dosimetric characteristics of several collimators appropriate for pMBRT. Comprehensive measurements are essential to thoroughly understand the pMBRT technique’s dosimetric properties. In addition to commonly used film measurements, high spatial resolution point detectors such as MicroDiamond and Razor diode detectors are also employed. Furthermore, extensive percent depth dose (PDD) curves are measured in solid water using various collimator designs, proton energy, and air gaps.

## 2 Materials and methods

### 2.1 Proton radiation unit

**Figure 1:**
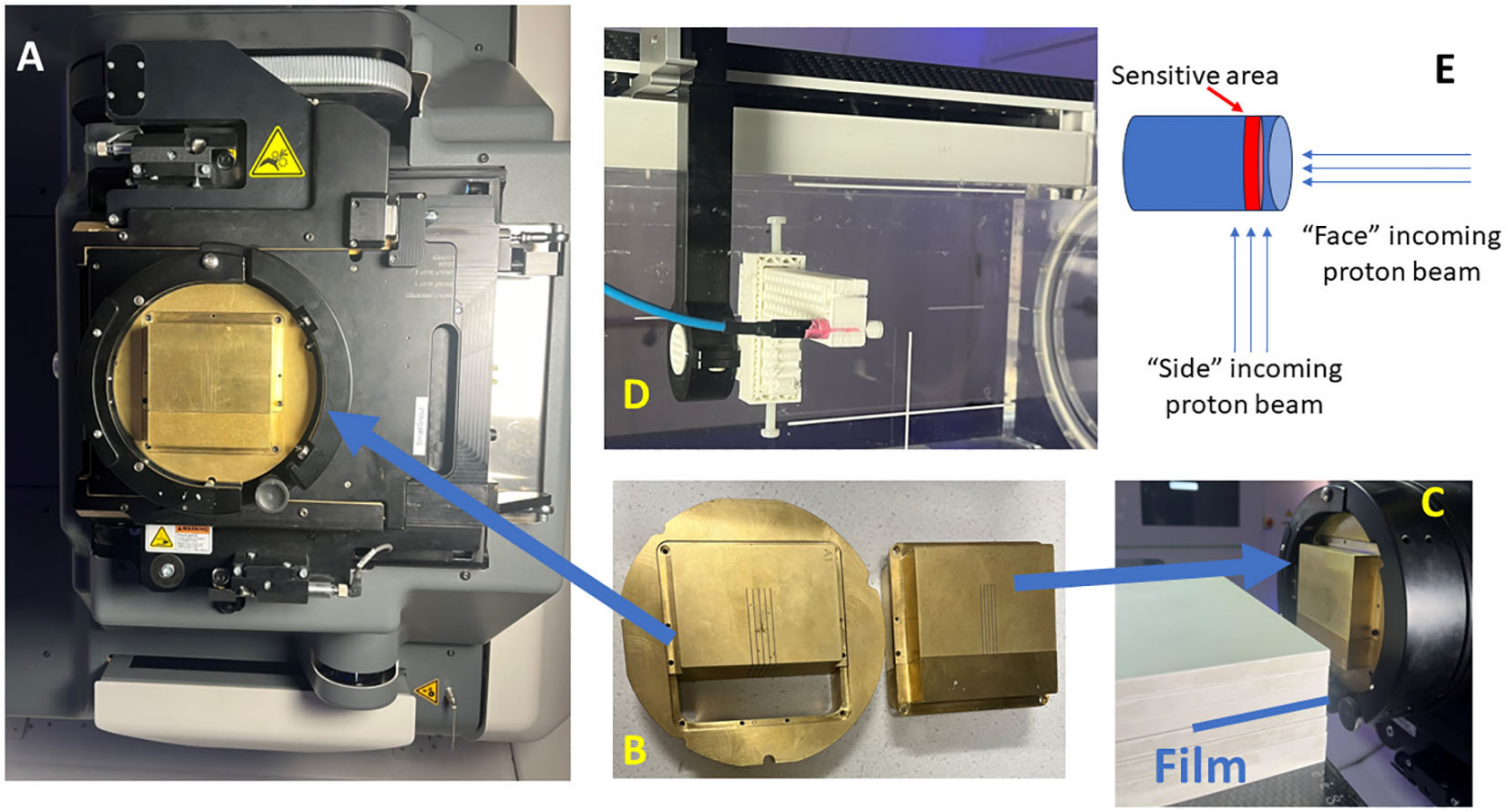
Proton minibeam radiation therapy (pMBRT) system. **(A)** The collimator is held by the snout accessory attached to the gantry. **(B)** The collimator is composed of three pieces. **(C)** Film measurements are set up for pMBRT PDD, where the film is stacked between the solid water layers. **(D)** The setup for the microDiamond and Razor diode measurements for the lateral profiles of pMBRT. **(E)** The schematic illustrates the orientation of the beam direction relative to the detector placement.

The detailed setup was described by Lin et al. (22). IBA Proteus®ONE (IBA, Louvain-La-Neuve, Belgium) is the proton machine used. The pMBRT collimator is mounted on a snout attached to the proton gantry as a beam-modifying accessory (see **Figure 1A**). The air gap can be adjusted by varying the snout position. Protons are delivered using a dedicated scanning beam nozzle. A unique feature of IBA Proteus®One is the relatively long source-axis-distance (SAD) in the patient’s superior and inferior directions, measured between 870 cm to 980 cm, averaging at 910 cm for 5x5 cm (2) field size. In the previous study, we demonstrated that for field sizes less than 2 cm, a parallel slit is sufficient to achieve better than 2% field uniformity. The collimator can be assembled and prepared for installation on the small snout with the air gap adjustable using the snout position. The irradiation field is generated in the RayStation treatment planning system (TPS) (Version 2023B, RaySearch Laboratories, Stockholm, Sweden), with all spots carrying a weight of 1 MU (Monitor Unit). The MU can be scaled individually per field during field delivery in the physics mode.

### 2.2 Collimators

The collimator designs have been previously described (22) (.decimal®, Sanford, FL, USA). In summary, the thickness of the collimator is 6.5 cm and is 5 cm in length direction. The collimators evaluated in this study are summarized in **Table 1**. Each collimator consists of three pieces, an outer ring to fit the snout slot, and the body split into two pieces to facilitate slit cutting using the Electrical Discharge Machining (EDM) technique, as shown in **Figure 1B**. The collimators are made from brass. The machining accuracy for EDM is generally approximately +/- 25 μm. All collimators in this study are machined with parallel slits aligned to the divergence to the long SAD direction (also referred to as Y direction from our previous work) (23).

**Table 1:** Summary of collimator properties.

Collimator ID	c-t-c (mm)	Slit (mm)	Number of slits	Lateral field size
1	2.8	0.4	5	5 cm x 1.1 cm
2	3.2	0.4	5	5 cm x 1.3 cm
3	4.0	0.4	5	5 cm x 1.6 cm

### 2.3 Radiation measurements using films

Gafchromic EBT-XD films are used for high spatial resolution dose measurements (24, 25) (Ashland, Bridgewater, NJ, USA). An Epson scanner, model Expression 11000XL (Epson America, Inc., Los Alamitos, CA, USA) was used for digitizing irradiated films. The EBT-XD films have a dynamic range of

0.4 Gy - 40 Gy. Film calibration was performed with 22 data points spaced between 0.4 Gy-40 Gy, using a 6cm x 6cm uniform proton field at the energy of 226MeV. A minimum valley dose of 1 Gy was maintained across all cases to ensure accurate valley dose measurements. Both calibration and measurement films were scanned 24 hours post-irradiation, with film orientation marked to ensure consistency. The scanning resolution is 300 dpi, corresponding to a pixel spacing of 0.8 mm. Scanned film analysis is performed using the IBA MyQA film panel application software, selecting the multi-channel analysis option within the MyQA framework. Additionally, film rotation adjustments can be made as needed under the MyQA patient QA analysis panel. The film is positioned proximal to or upstream of the microDiamond and Razor diode detectors for lateral dose profile validation and aligned perpendicular to the beam direction. Subsequently, the film dose is extracted and compared with the measured dose of the micoDiamond and Razor diode detectors.

The irradiation map consists of a uniform proton field with a single energy layer covering 5 cm x 2 cm, with 5 mm spot spacing at the isocenter. The pMBRT is generated when these uniform fields pass through the multi-slit collimator. **Table 2** lists all the measurement scenarios. Section 2.4 provides detailed information regarding the microDiamond and Razor diode detector measurements.

**Table 2:** Summary of dosimetry measurements for various pMBRT collimators.

Collimator ID: #1 Slit width: 0.4 mm, center-to-center distance: 2.8 mm	
Films for lateral profiles	Depths of these lateral profiles taken: 1 cm, 4.5 cm, and 6.5 cm Proton Energy used: 150 MeV Air gap used: 6 cm
microDiamond	
Razor diode	
Films for depth profiles	Air gap at 6 cm, proton energy used: 70, 80, 100, 120, 150, and 180 MeV
	Proton energy used 150 MeV, air gap used: 2, 4, 6 and 8 cm

Collimator ID: #2 Slit width: 0.4 mm, center-to-center distance: 3.2 mm	
Films for lateral profiles	Depths of these lateral profiles taken: 1 cm Proton Energy used: 150 MeV Air gap used: 6 cm
microDiamond	
Films for depth profiles	Proton energy used 150 MeV, air gap used: 6 cm

Collimator ID: #3 Slit width: 0.4 mm, center-to-center distance: 4.0 mm	
Films for lateral profiles	Depths of these lateral profiles taken: 1 cm Proton Energy used: 150 MeV Air gap used: 6 cm
microDiamond	
Films for depth profiles	Proton energy used 150 MeV, air gap used: 6 cm

For depth dose validation, the films are stacked between solid water phantoms (SP34 QA Phantoms, IBA Dosimetry, Schwarzenbruck, Germany), with the film positioned parallel to the beam direction, as shown in **Figure 1C**. This positioning could potentially lead to air pockets along the beam direction. To prevent this, the couch is tilted +2 degrees in roll according to the IEC 61217 coordinate standard, and clamps are also used to compress the solid waters, ensuring the air pockets are minimized. The PDD measurements with an air gap of 6 cm are conducted using a mono-energy layer of proton beams with energies of 70 MeV, 80 MeV, 100 MeV, 120 MeV, 150 MeV, and 180 MeV.

### 2.4 Radiation measurements using diamond detectors and diodes

In addition to Gafchromic films, the microDiamond (TM60019) detector (PTW, Freiburg, Germany) and Razor diode detector (IBA Dosimetry, Schwarzenbruck, Germany) are utilized for pMBRT dosimetry validation (21, 26, 27). The detectors are cross-calibrated with a PPC 05 ionization chamber (IBA Dosimetry, Schwarzenbruck, Germany) using a 226 MeV proton beam at a depth of 2 cm within a 10 cm x 10 cm square proton field. Thecalibration process is performed with the side of the detector facing the incoming beam (**Figure 1E**), consistent with the orientation used for most of the measurements in this study. Additionally, the cross-calibration is conducted with the circular face of the microDiamond detector facing the incoming beam, revealing a difference of less than 0.5%. Due to the minimum discrepancy observed, corrections were not applied to readings when the detector faced the beam. The detectors are mounted on the Blue Phantom PT scanning arm using a 3D printed adapter, as shown in **Figure 1D**. Lateral profiles at various depths are measured using additional solid water pieces. A film is also positioned proximal to or upstream of the detectors for cross-validation. A uniform proton field with dimensions of 5 cm x 2 cm and 5 mm spot spacing is delivered at each measurement point. The measurement step size is 0.1 mm near the peak and valleys and 0.2–0.3 mm elsewhere along the lateral profiles. The microDiamond detector has an active measurement area with a thickness of 0.001 mm, and the Razor diode has a thickness of 0.02 mm. The diameter of the active measurement area is 4 mm for the microDiamond detector and 1 mm for the Razor diode. During lateral profile measurements, the thin side of the detectors is scanned across the lateral profiles. Additionally, measurements are conducted with the microDiamond detector facing the beam to capture data from this orientation as well while scanning across the lateral profiles.

To compare the lateral profiles, 1D gamma analysis is employed to compare lateral dose curves obtained from different radiation detectors. In conventional clinical applications, search distances between 1–3 mm are commonly used. However, pMBRT involves rapid dose changes at the submillimeter level, necessitating a more sensitive approach. Therefore, we use 1D gamma analysis criteria of 3%/0.2 mm and 3%/0.1 mm, which are aligned with the resolution needed to resolve the dose heterogeneity. For this 1D gamma analysis, the comparison is limited to distances up to 2.0 mm from the most lateral dose peak without applying a threshold dose cut-off. This approach comprehensively evaluates the agreement between measured and reference dose distributions.

### 2.5 Monte Carlo simulations

The Monte Carlo (MC) simulations for the study were conducted using TOPAS (23, 28, 29). Detailed information about the Monte Carlo simulation process can be found in our previous work (23). In summary, The TOPAS build version is 3.9. The simulation phantom consisted of water with dimensions of 8 cm x 8 cm x 30 cm. The beam model was tailored to match our clinical beam parameters, as described in previous publications (23, 28). The physics modules included are (g4em-standard_opt4, g4h-phy_QGSP_BIC_HP, g4decay, g4ion-binarycascade, g4h-elastic_HP, g4stopping). The MC simulations are performed to calculate the 3D dose distribution in water, which are subsequently compared with film-based PDD measurements. The scoring dose grid was set to 0.5 mm in the depth and slit length direction and 0.1 mm perpendicular to the slit direction. This adaptive, non-uniform scoring grid was chosen to accurately capture the spatial resolution necessary for spatially modulated heterogeneous dose distributions.

### 2.6 Open-field PDD measurements with film for LET correction

For depth dose measurements of particle therapy using films, the impact of LET dependency has been observed, leading to a tendency for underestimated dose reading near the end of the range due to the increasing LET (30–32). This phenomenon indicates the importance of accounting for LET effects when interpreting film-based dosimetry in proton therapy. In our study, we address the LET dependency observed in mono-energetic depth dose measurements using films by implementing corrections for the LET dependency near the end of the range. We have adapted the approach described by Peucelle C. et al., where corrections are applied to account for LET-induced dose changes at the end of the proton range through an open-field irradiation (33, 34). This correction method ensures that film-based dosimetry provides a more accurate representation of the dose distribution across the entire depth dose curve.

For the open field irradiation, the film irradiation configuration mirrors the PDD pMBRT irradiation, and the setup is shown in **Figure 1C**. The film is positioned along the beam direction, with the couch tilted +2 degrees in roll to minimize air pockets. No other beam-modifying devices are placed in the beam direction. The open field films were irradiated using a mono-energy layer of proton beam with a field size of 5 cm x 5 cm and energies of 70 MeV, 80 MeV, 100 MeV, 120 MeV, 150 MeV, and 180 MeV. This setup ensures consistent conditions for measuring depth dose characteristics across various proton energies.

**Figure 2:**
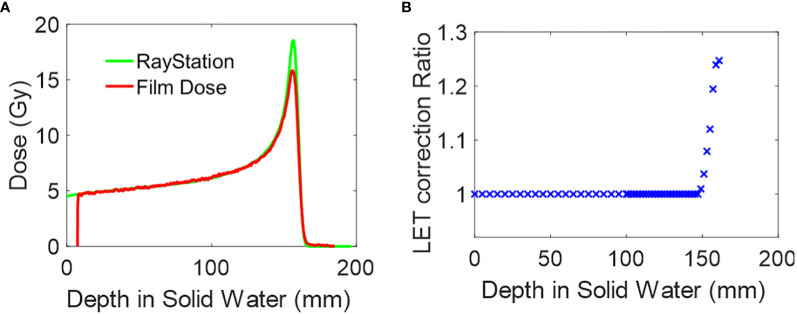
Film dosimetry LET correction. **(A)** Depth dose plot of 150MeV mono-energy proton field calculated in RayStation and measured by film. The curve is matched at D90. **(B)** The LET correction ratio is derived from **(A)**.

The dose for open-field, single-energy layer proton dose is calculated using the clinical treatment planning system, RayStation. The computed dose is then compared with the dose derived from film measurements, as shown in **Figure 2A**. Both PDD curves are matched at D90, which is defined as 90% of the maximum dose. A depth-dependent LET correction ratio is obtained by dividing the two curves, as illustrated in **Figure 2B**. Two key considerations are made for generating the correction ratio curve, as shown in **Figure 2B**. First, the correction ratio solely addresses LET dependency in film dosimetry. The ratio is recorded only when it exceeds a threshold of 1.005, and any correction value proximal to that depth will be set to 1. Secondly, to reduce noise, the TPS dose was calculated with a Monte Carlo dose calculation uncertainty of 0.1%, and the film dose was extracted by averaging the central 5 pixels along the PDD curve direction. The process is repeated for each mono-energy setting used in the pMBRT PDD measurements, as outlined in **Table 2**.

## 3 Results

### 3.1 Lateral profile measurements of pMBRT

**Figure 3:**
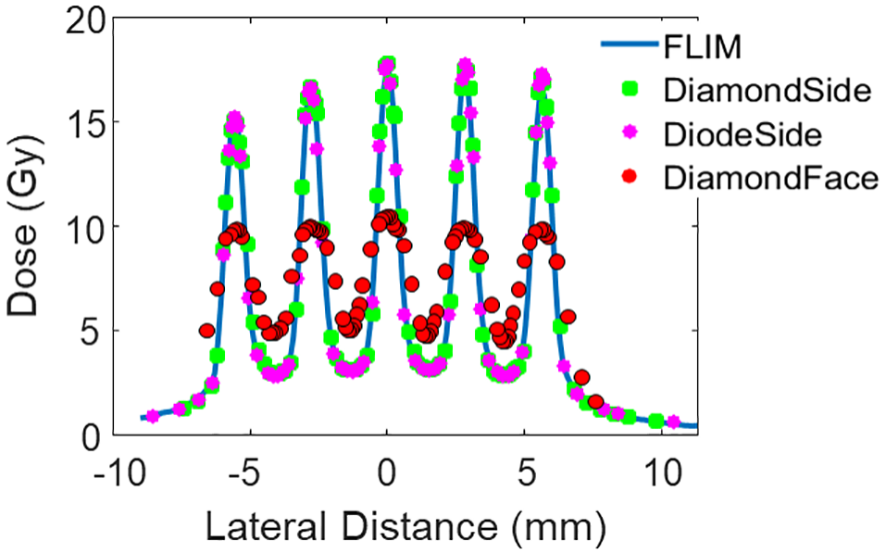
Lateral dose profile of Collimator #1 at measurement depth 1 cm. The collimator has 5 slits, a slit width of 0.4 mm, and a c-t-c of 2.8mm.

**Figure 4:**
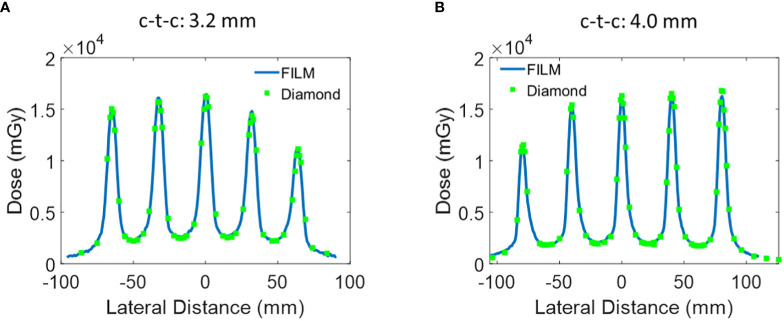
Additional lateral dose profile of Collimator #2 and #3 measured at a depth of 1 cm. **(A)** Collimator #2 has 5 slits, slit open of 0.4 mm, and c-t-c of 3.2 mm. **(B)** Collimator #3 has 5 slits, a slit width of 0.4 mm, and a c-t-c of 4.0 mm.

**Figure 5:**
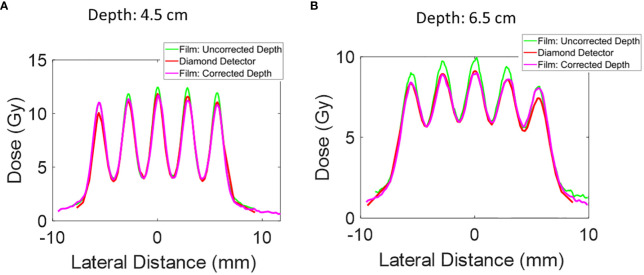
The lateral dose profile for Collimator #1 was measured at additional depths of 4.5 mm and 6.5 mm. The uncorrected depth represents the proximal end of the microDiamond detector, and the corrected depth represents the depth of the center of the microDiamond detector. The collimator has 5 slits, a slit open of 0.4 mm, and a c-t-c of 2.8mm. **(A)** Lateral dose profile at depth 4.5 cm. **(B)** Lateral dose profile at depth 6.5 cm.

The dose measurements using EBT-XD film, microDiamond PTW detector, and Razor diode detector are presented in **Figures 3**–**5**. This section includes all the film measurements for lateral profiles, as detailed in **Table 2**. Lateral profiles for aperture ID #1 were measured at depths of 1 cm, 4.5 cm, and 6.5 cm, while those for aperture ID #2 and #3 were measured at a depth of 1 cm. For these measurements, a proton energy of 150 MeV was used, with a collimator air gap at 6 cm.

Additional dose measurements were performed at a depth of 1 cm using the microDiamond detector orientated with the detector’s active area facing the beam. The average doses obtained from the four measurements are 7.36 Gy using EBT-XD film, 7.52 Gy using the microDiamond detector, 7.41 using the Razor diode detector, and 7.56 Gy using the microDiamond detector facing the beam direction. The average dose is extracted by averaging over four center valley areas, covering the central lateral distance of three times of the c-t-c distance.

The red dotted curve in **Figure 3** illustrates that the small active area of microDiamond diameter is insufficient to capture the dose variations accurately in the sub-millimeter pMBRT pattern. Despite this limitation, the average dose measured by the microDiamond detector when oriented facing the beam is 7.56 Gy, which is consistent with the measurements obtained from other high-resolution detectors, with a variance within 2%.

The 1D gamma analysis comparing the microDiamond detector (with the thin side aligned with the beam) and the Razor diode detector to the film curve reveals a passing rate of 99% for both detectors at the 3%/0.2 mm criteria. The passing rate is 98% for the microDiamond detector and 99% for the Razor diode detector at the 3%/0.1 mm criteria. As expected, the microDiamond detector, when facing the beam direction, showed a significantly lower passing rate of 34% using the 3%/0.2 mm criteria.

The lateral dose measurements using EBT-XD film, microDiamond PTW detector were then repeated for the other two collimators with c-t-c distance of 3.2 mm and 4.0 mm. Similar to the c-t-c 2.8 mm collimator, the film and microDiamond measurements were in agreement. The 1D gamma analysis comparing the microDiamond curve to the film curve revealed a passing rate of 100% at 3%/0.2 mm criteria for both Collimator #2 and Collimator #3. The passing rate slightly decreased to 97% and 98% when the criteria were set to 3%/0.1 mm for Collimator #2 and Collimator #3, respectively.

Further depth measurements were conducted with Collimator #1 at 4.5 cm and 6.5 cm depths. The film positioned directly upstream of the microDiamond detector showed a higher peak dose than the microDiamond detector measurements. To investigate this discrepancy, we extracted the lateral dose profile from the film measurement for the PDD curve at depths of 4.7 cm and 6.7 cm to account for the diameter of the diamond detector. After this correction, the lateral profile measurements were more consistent with the microDiamond detector measurements.

At a depth of 4.5 cm, the 1D gamma analysis comparing the microDiamond curve to the corrected film curve reveals a passing rate of 98% at 3%/0.2 mm criteria. In contrast, the uncorrected film data gives a passing rate of 87%. At a depth of 6.5 cm, the 1D gamma analysis shows a passing rate of 92% at 3% 0.2 mm criteria for the corrected film data, while the uncorrected film data results in a passing rate of 66%. This highlights the importance of accounting for detector dimensions in high-resolution dosimetry.

As shown in **Figure 2A**, conventional open-field proton beam PDD gradually increases in the entrance plateau region and rises sharply as the protons slow down and deposit dose rapidly at the end of the range, creating the pristine Bragg Peak. In contrast, the pMBRT PDD exhibits a distinct pattern: the peak region initially decreases before rising near Bragg Peak, while the valley region initially demonstrates an increase in dose. After correcting for the dose profile extracted at deeper depths, the film peak dose is observed to be lower, as shown in **Figure 5**.

### 3.2 MC validation of PDD with various air gaps, with/without LET correction

Maintaining a consistent and reproducible air gap setup is crucial for accurate pMBRT dosimetry, as highlighted in prior studies (21, 22). In our measurements, PDD curves of these pMBRT collimators were extracted using film positioned between the layers of solid water and aligned parallel to the beam’s incoming direction, as described in section 2.3 and shown in **Figure 1C**. Given the dose variations characterized by peaks and valleys in the lateral profile direction, the peak PDD was determined by identifying the highest peak value in the depth profile. Conversely, the valley PDD is extracted by averaging the values of the two adjacent lowest peak depth profiles. Additionally, to ensure consistency and accuracy in our measurements, film orientations in the depth direction were adjusted using the rotation feature within the MyQA software’s patient QA module.

**Figure 6:**
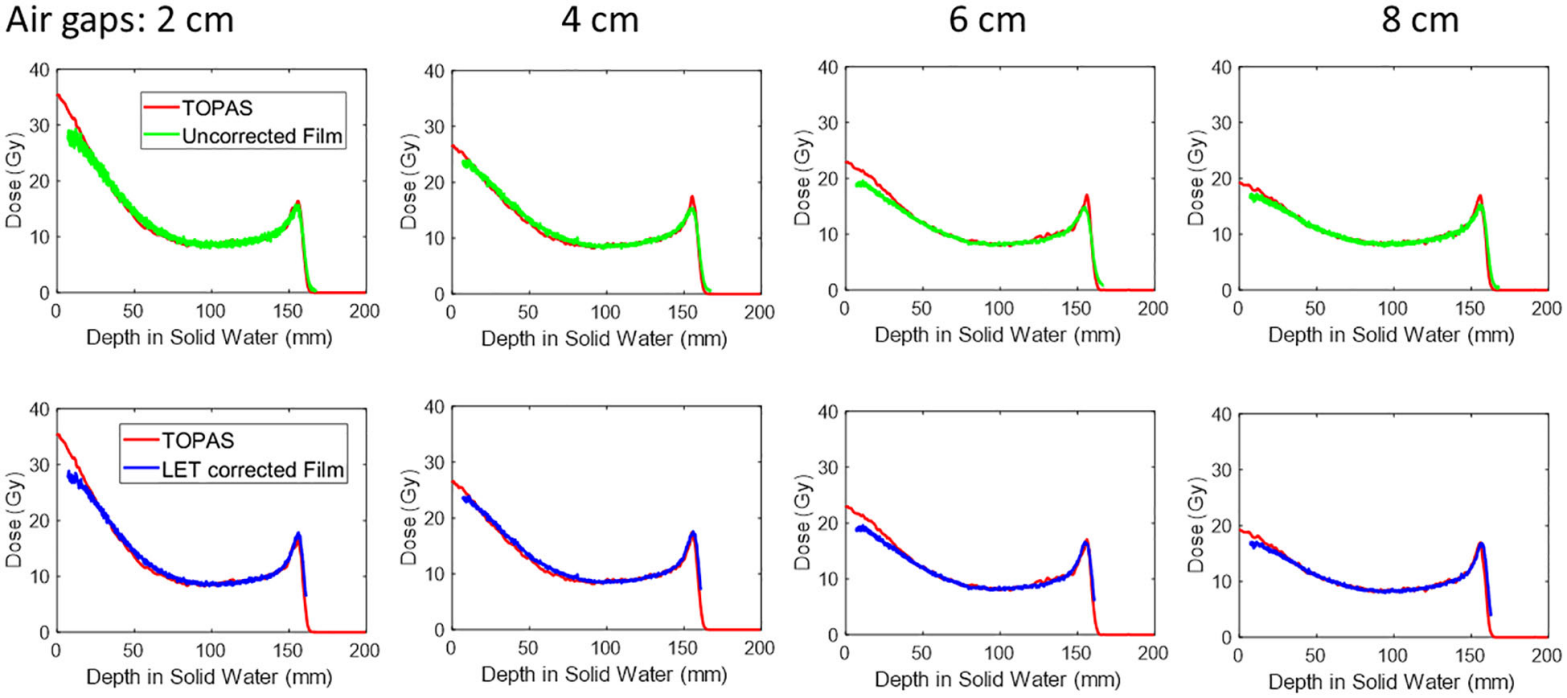
The peak PDD of 150MeV mono-energy beam for Collimator #1. This is measured using various air gaps, 2 cm, 4 cm, 6 cm, and 8 cm. The top panel shows the film dose without LET correction, and after correction, the curve is shown in the lower panel.

The peak PDD of 150 MeV mono-energy beam for Collimator #1 with various air gaps is shown in **Figure 6**. To demonstrate the necessity of correcting for the LET dependency of the film, we compared the uncorrected PDD curve derived from the film to the results from TOPAS simulation in the top panel. It was observed the peak region was underestimated by 23%. However, after applying the correction using the depth correction curves outlined in Section 2.6 and shown in **Figure 2B**, the dose extracted from the film is closely aligned with the TOPAS simulation across the entire depth curve.

For instance, at a depth of 1 cm, the peak dose reduced from 31.3 Gy to 18.1 Gy as the air gap increased from 2 cm to 8 cm. Similarly, at a depth of 5cm, the dose decreased from 13.7 Gy to 11.5 Gy with the same air gap variation.

### 3.3 Modulation of PVDR in pMBRT with various air gaps

**Figure 7:**
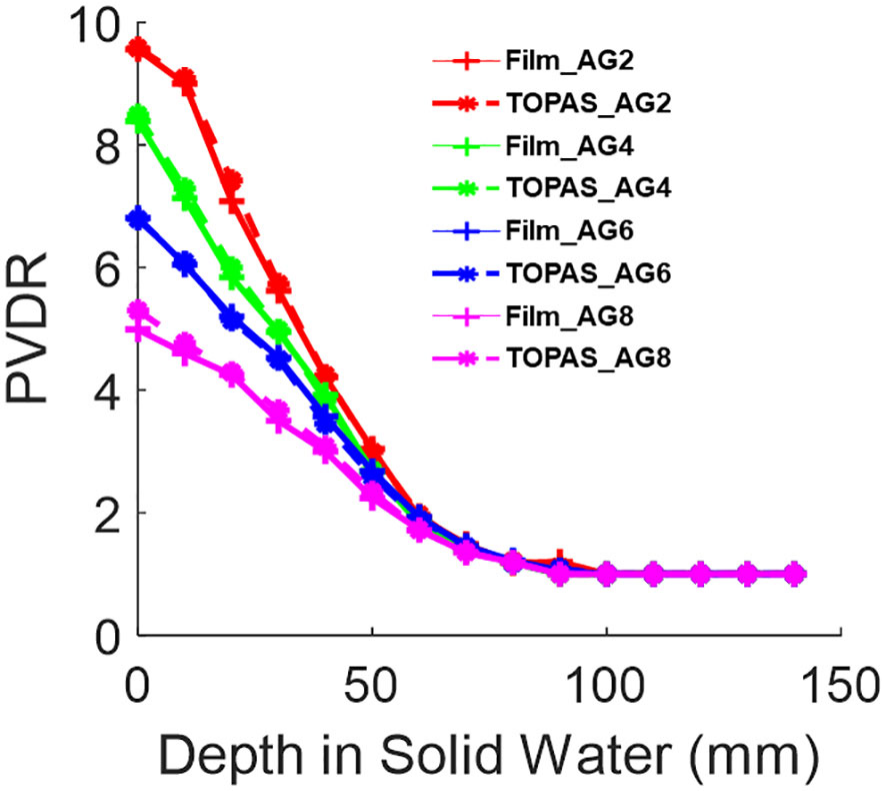
The PVDR of 150MeV mono-energy beam for Collimator #1. This is measured using various air gaps, 2 cm, 4 cm, 6 cm, and 8 cm, shown in the legend as AG2, AG4, AG6, and AG8, respectively. The film measurements are compared against TOPAS MC simulations.

**Figure 7** shows the PVDR of various air gaps for Collimator #1 with 150 MeV proton beams. At a depth of 1 cm, the PVDR is 9.1 when the air gap is 2 cm. As the air gap increased to 8 cm, the PVDR decreased to 4.8. This demonstrates that adjusting the air gap can serve as an additional parameter to achieve different PVDRs during the design of pre-clinical studies.

### 3.4 PDDs of pMBRT with various proton energies

**Figure 8:**
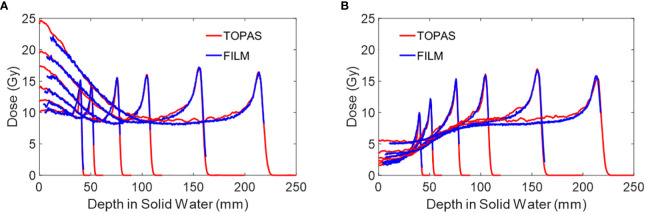
**(A)** The peak PDD of several mono-energy beams for Collimator #1, using air gap of 6 cm. Energies used are 70 MeV, 80 MeV, 100 MeV, 120 MeV, 150 MeV, and 180 MeV from left to right. **(B)** The valley PDD of several mono-energy beams for Collimator #1, using an air gap of 6 cm. Energies used are 70 MeV, 80 MeV, 100 MeV, 120 MeV, 150 MeV, and 180 MeV from left to right. The PDD measurements by film are LET corrected and are compared against TOPAS MC simulations.

**Figure 8** shows the peak and valley region PDD for several mono-energy beams. Each curve includes LET correction, which is applied based on its respective open-field film measurements. **Table 3** shows the PVDR of each energy versus depth.

**Table 3:** PVDR for various energy using Collimator #1.

Depth	E70		E80		E100		E120		E180	
	TOPAS	FILM	TOPAS	FILM	TOPAS	FILM	TOPAS	FILM	TOPAS	FILM
1 cm	5.76	5.58	6.17	6.09	6.29	6.02	6.32	6.29	4.90	4.84
2 cm	4.43	4.32	4.88	4.78	5.14	5.27	5.20	5.20	4.33	4.39
3 cm	2.76	2.84	3.12	3.22	4.01	4.06	4.18	4.18	3.83	3.82
4 cm	1.66	1.69	1.84	1.89	2.37	2.48	3.05	3.10	3.62	3.53
5 cm			1.34	1.31	1.67	1.68	1.98	2.10	2.79	2.82
6 cm					1.21	1.24	1.46	1.52	2.27	2.22
7 cm					1.05	1.05	1.17	1.18	1.62	1.69
8 cm									1.38	1.37
9 cm									1.15	1.17
10 cm									1.08	1.08

### 3.5 PDDs of pMBRT with various collimators

**Figure 9:**
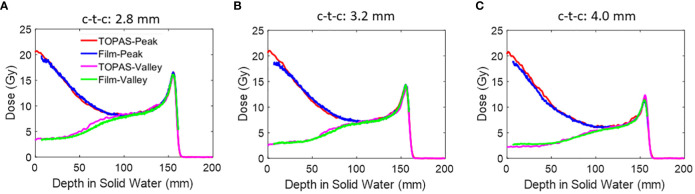
**(A)** The peak and valley PDD of 150 MeV beams using an air gap of 6 cm. The PDD measurements by film are LET corrected and are compared against TOPAS MC simulations. Three collimators are presented here. **(A)** Collimator #1; **(B)** Collimator #2; **(C)** Collimator #3.

**Figure 9** shows the PDD of the peak and valley regions of the three collimators using 150 MeV, with LET correction applied. The PVDRs are listed in **Table 4**. The air gap for all the measurements is 6 cm. The values under the “Collimator 1” column in **Table 4** were also utilized for plotting **Figure 7**, where the air gap was set to 6 cm.

**Table 4:** PVDR for three collimators at various depths. .

Depth	Collimator 1		Collimator 2		Collimator 3	
	TOPAS	FILM	TOPAS	FILM	TOPAS	FILM
1 cm	6.11	6.11	7.56	7.38	8.42	8.55
2 cm	5.00	4.92	6.37	6.37	7.44	7.59
3 cm	4.38	4.43	5.46	5.56	6.68	6.66
4 cm	3.55	3.53	4.52	4.57	5.47	5.58
5 cm	2.60	2.79	3.43	3.49	4.55	4.53
6 cm	1.94	2.06	2.36	2.51	3.47	3.31
7 cm	1.51	1.55	1.78	1.94	2.48	2.55
8 cm	1.19	1.22	1.50	1.53	1.85	1.95
9 cm	1.08	1.12	1.16	1.20	1.52	1.49
10 cm	1.0	1.0	1.06	1.09	1.32	1.34
11 cm			1.0	1.0	1.10	1.11
12 cm					1.0	1.0

## 4 Discussion

In this study, we present comprehensive dosimetric methods for pMBRT measurements. pMBRT poses significant dosimetric challenges due to its highly heterogeneous lateral dose distribution and rapidly varying dose distribution in the depth direction. We examined pMBRT multi-slit collimators with three different center-to-center distances across various proton energies and air gaps. Film measurements were rigorously validated and compared against high-resolution detectors such as microDiamond and Razor diode detectors. This approach enhanced confidence in commissioning pMBRT as the doses are validated with multiple radiation detectors. Furthermore, all film measurements were benchmarked against Monte Carlo simulation, offering additional confidence in the dosimetric validation of pMBRT.

As pointed out in previous works, considering beam divergence is important for pMBRT (18, 22, 34). The IBA Proteus®ONE system has a relatively long SAD along the patient superior-inferior (SI) direction, averaging at 910 cm (23). This specific characteristic has been extensively investigated in our previous work (23). Our simulation studies have demonstrated the uniformity of the parallel slits remains better than 2% in the SI direction. Therefore, the collimators in this study are aligned parallel to the patient’s SI direction, ensuring consistent measurements with slits oriented to experience minimum divergence. Due to the small open size of the pMBRT slit (0.4 mm) across 6.5 cm thick brass metal, the machining accuracy can vary by about 10% (resulting in a 40 μm uncertainty), which exceeds the uniformity loss from beam divergence. In this work, we observed differences in the peak dose values of all dose profiles, particularly at the outmost peaks, as shown in **Figures 3**–**5**. This discrepancy is not solely due to the beam divergence, which theoretically only accounts for less than 4% of the peak dose differences (18, 21). Instead, the primary limitation arises from our current collimator design (see **Figure 1B**). The 5 cm long slits, made of soft brass material, can potentially be compressed, causing the side slit to distort. The compression might introduce an uncertainty of 40–100 μm, which can be negligible for slits wider than 2 mm. However, for the 0.4 mm opening size in our pMBRT collimator, this uncertainty can exceed 10%, leading to a peak dose loss of more than 10%. We are currently working on improving the design to further increase the rigidity and consistency of all the slits across the collimator. Despite the peak dose variations, our study demonstrates the consistency of various high spatial resolution dosimetric devices for the commissioning measurements of pMBRT. Due to the use of 6.5 cm thick brass metal, achieving machining accuracy better than 10% dose variations might be challenging. This necessitates the future development of a pMBRT dose calculation TPS that can account for individual slit variations.

The measurement uncertainty comes from two primary sources. The first source is setup uncertainty, which includes factors such as collimator assembly, daily gantry and couch setup accuracy, and air gap consistency. These aspects have been discussed in our previous work, where we demonstrated a consistency within 5% for all repeats and various gantry angles (23). The second source of uncertainty comes from the measurement devices and the consistency of the machine output. The Proteus®ONE system has an intrinsic dose uncertainty of +/- 1%, with irradiation stops when the MU count falls within 1% of the total MU requested per field. Daily observations indicate that the output variation follows a normal distribution and typically remains below 0.5%. For the microDiamond and Razor diode detectors, we ensure the measurements are stable within 2% before proceeding with the full measurements. This approach helps to minimize uncertainties and improve the reliability of the dosimetric data. Every 5 measurements, we check if this condition is still met. pMBRT measurements using PBS are particularly time-consuming, as each 0.1–0.2mm movement along the lateral distance requires a full field to be delivered, taking several hours for one profile at a given depth. For film measurements, we have refined the dose reading process by ensuring the following: 1. Films are scanned at a consistent location on the scanners. 2. Films are scanned consistently 24 hours post-irradiation. 3. The calibration process is repeated every three months to ensure film quality remains unchanged. The film results presented in **Figure 3** include five technical repeats, with the last three repeats showing measurement uncertainty controlled within 5%. We observed nearly a 10% dose difference with incorrect film orientation and more than a 5% dose difference if scanned within 10 hours post-irradiation. This study does not show these specific results as they reflect a well-documented phenomenon in film dosimetry (24, 25).

In pMBRT research, the TOPAS Monte Carlo tool is frequently used to validate experimental results (18, 26, 28, 35, 36). The flexibility and versatility of TOPAS make it an ideal tool for introducing and implementing new techniques. However, to advance pMBRT toward clinical trials, developing a dose calculation protocol on a commercial treatment planning system (TPS) platform will be essential.

The agreement of the PDD curves between experimental film measurements and TOPAS Monte Carlo simulation shows some discrepancy, particularly in the first 2–3 cm. Unfortunately, limited published comparison work compares experimental and MC dose calculations for pMBRT PDD curves. Most pMBRT studies have been carried out with slit widths greater than 1 mm (36–38). The most extensive pMBRT work with submillimeter slit widths has been carried out by the research group from Institut Curie-Orsay proton therapy center. Although most of the commissioning results focused on lateral profiles, Ludovic De Marzi et al., provided a pMBRT PDD plot using Razor diode and MC calculations with 5cm x 5cm, 123 MeV,150 MeV, and SOBP proton beam for collimators with a 0.4 mm slit width. Discrepancies were also observed in the first 2–3 cm (18). There are several potential reasons for the observed discrepancy. First, our TOPAS Monte Carlo simulation models the proton beam as a Gaussian shape, which may not fully account for the halo dose produced in the beamline (28). This halo effect can be more pronounced at lower energies and shallower depths (18, 39). Second, efforts were made to reduce the air pockets in the film measurements of PDD, particularly since the beam direction is parallel to the film positioning direction. The measures taken to reduce air pockets included tilting the table by two degrees and using mechanical clamps to compress the solid water slabs physically. Despite these efforts, shallow doses may still be susceptible to residual effects. Additionally, the machining of the slit width by the company may introduce uncertainties up to 10% (40 μm uncertainty), potentially affecting the peak dose profile.

In this work, we addressed the issue of LET dependency in film measurements through open-field film measurements to apply LET correction. However, challenges can arise when measuring spread-out Bragg peak (SOBP) using films, particularly in correcting LET with mixed energies (31, 32). This will be a focus of future studies. In section 3.2 and **Figure 6**, we presented both corrected and uncorrected PDD curves for pMBRT regarding LET. The curves include the original raw measurements without correction for two primary purposes. First, the uncorrected curve provides additional raw data directly extracted from the film, facilitating comparisons for future studies on the topic of pMBRT dosimetry. Secondly, if a pMBRT dose is not intended for use in the high LET region for pre-clinical studies, films can be used for dosimetry without correction, provided proper calibration is performed.

Certainly, the microDiamond and Razor detectors excel in high-precision point measurements, but their use in minibeam dosimetry remains challenging, motivating further improvement and research in this area. The microDiamond detector is exceptionally suited for clinical stereotactic radiosurgery (SRS) due to its capability to measure small field sizes, such as those from SRS cones with diameters as small as 4 mm. However, its use in pMBRT, which features submillimeter slit width (0.4 mm slit opening in our case), remains challenging. As we showed in **Figure 5**, the rapidly changing lateral dose profile across depth, necessitates corrections for the microDiamond’s own diameter thickness. This adjustment is needed to ensure accurate measurements in the context of pMBRT.

Due to similar constraints, 2D high-resolution detectors also face challenges, prompting the frequent use of Gafchromic film known for its high dynamic range, as the primary dosimetry tool. However, addressing the LET dependency in film measurements remains challenging, with its intrinsic uncertainty needing to be addressed before the clinical implementation of pMBRT.

## 5 Conclusion

In this work, we have presented comprehensive dosimetric measurements for pMBRT. Our findings demonstrate excellent agreement among various dosimetry devices when properly calibrated and corrected. This indicates that the pMBRT technique has the capability to achieve diverse PVDR under different conditions.

## Data availability statement

The raw data supporting the conclusions of this article will be made available by the authors, without undue reservation.

## Author contributions

YL: Conceptualization, Data curation, Formal analysis, Investigation, Methodology, Resources, Software, Supervision, Visualization, Writing – original draft, Writing – review & editing. WL: Data curation, Formal analysis, Software, Validation, Writing – original draft. AW: Data curation, Investigation, Writing – original draft. DJ: Data curation, Methodology, Writing – original draft. GG: Conceptualization, Investigation, Writing – original draft. HG: Conceptualization, Formal analysis, Funding acquisition, Investigation, Project administration, Resources, Supervision, Validation, Visualization, Writing – original draft, Writing – review & editing.
